# The Pathology of Recurrent Appendicitis

**Published:** 1897-09

**Authors:** 


### THE PATHOLOGY OF RECURRENT APPENDICITIS.

Whether every case of appendicitis should be treated surgically or not, even when the most approved medical measures have failed to effect speedy relief, and whether or not the diseased appendix should be removed invariably, are still debatable questions. There can be no doubt that in many instances recovery, from the immediate attack at least, ensues without surgical intervention,and it is even probable that some attacks pursue a favorable course without so much as being recognized, though less commonly at present than in the not remote past. Death from peritonitis of obscure origin also is less common now than formerly.

When, however, one reflects upon the frequency with which an attack of appendicitis is followed by others when the diseased organ is permitted to remain, it becomes an exceedingly delicate matter to decide in the individual case whether it is better to undertake a radical operation or to submit to the uncertain risks and dangers that attend a lesion of undeterminable extent and degree. While the mortality from the disease may not be greater in competent medical than in equally competent surgical hands, the future of the case appears assuredly less certain under the former than under the latter conditions. The final decison, however, in favor of the one or the other course of procedure will depend ultimately upon a thorough appreciation of the pathology and the natural history of the morbid process.

It is for this reason that a recent report by Southan,* of the pathological conditions found in twenty cases of recurrent appendicitis treated by operation will be received with especial interest. Of this number seven occurred in females and thirteen in males. The youngest patient was ten, the oldest forty-four years of age. In fifteen of the cases the patients were between fifteen and thirty years of age. In all of the specimens examined the appendix showed evidences of chronic inflammatory changes with thickening of its coats. In some cases its lumen was uniformly narrowed and almost obliterated; in others it was partially or completely occluded at some point and dilated on the distal side of the obstruction, occasionally forming, when the occlusion was complete, a cystic cavity of some dimensions. In many instances the appendix was considerably shortened; it was frequently found bent upon itself and bound down by adhesions, in one case the tip almost touching the cæcal end of the process.

The contents of the appendix consisted either of clear mucus or of a muco-purulent fluid; in two cases a hard fæcal concretion was present in its interior, and in another a concretion which had ulcerated through its wall was found in an abscess cavity external to it. In most cases the inflammatory changes were not confined to the appendix itself, for evidences of appendicular peritonitis were generally found to be present, the peritonitis being usually of the adhesive variety, the inflam-

* The Lancet, June 5, 1897.

matory exudation that had been poured out round the appendix having undergone organization and forming adhesions. These were often very firm and extensive, surrounding the appendix and fixing it to the parietal peritoneum, omentum, cæcum, or small intestine. In some instances they were present after a second attack; in other instances they were absent after many attacks. When exceptionally dense and extensive, complete obstruction of the bowel may be produced from inclusion and compression of a small coil of small intestine in the adhesions, and if the condition is incapable of relief by operation the result is necessarily fatal.

In some instances the peritonitis was of the suppurative character, pus having formed in the neighborhood of the appendix. This complication was encountered in eight of the twenty cases. In six of these the suppuration was localized, an encysted intraperitoneal abscess being present. In two cases the suppuration was general, there being well-marked evidences of diffuse purulent peritonitis. When suppuration takes place it is often secondary to ulceration and perforation of the walls of the appendix, followed by escape of its contents. In one case the appendix, though not actually perforated, was extremely thinned at one point which was found to correspond with an ulcer involving its mucous and muscular coats.

Among the twenty cases the duration of the symptoms ranged from four months to six years, and the number of attacks from two to ten or more. If cases of recurrent appendicitis are left to themselves attacks may recur at irregular intervals for years, and ultimately a cure may take place by a gradual process of obliteration of the lumen of the appendix and its conversion into a fibrous cord. Suppuration may, however, occur at any time; if an encysted abscess forms, the appendix should, after evacuation of its contents, usually shrivel up, becoming obliterated and causing no further trouble. On the other hand, if the suppuration is diffuse—that is, if there is general purulent peritonitis—the result will probably be fatal. Another complication, already mentioned, that may occur is intestinal obstruction from compression of a coil of bowel by adhesions.

Of the various theories advanced to explain the occurrence of appendicitis, the most plausible is that which regards the appendix as a diverticulum that readily allows of the accumulation and stagnation of fæcal matter. This, mingling with the secretion from its mucous lining and undergoing fermentative or putrefactive changes, sets up a catarrhal inflammation which may be followed by ulceration and perforation or by thickening of its walls, the latter condition being that which is most commonly encountered in the recurrent form of the disease. The fæcal concretions found in the interior are probably the consequence and not the cause of the inflammation, being due to inspissation of its contents; but once formed, they no doubt tend to excite and keep up the recurrent attacks.—Editorial, *New York Medical Record.*

